# HIV-1 replication in cell lines harboring INI1/hSNF5 mutations

**DOI:** 10.1186/1742-4690-3-56

**Published:** 2006-08-31

**Authors:** Masha Sorin, Eric Yung, Xuhong Wu, Ganjam V Kalpana

**Affiliations:** 1 Department of Molecular Genetics, Albert Einstein College of Medicine, Bronx, New York, NY, USA

## Abstract

**Background:**

INI1/hSNF5 is a cellular protein that directly interacts with HIV-1 integrase (IN). It is specifically incorporated into HIV-1 virions. A dominant negative mutant derived from INI1 inhibits HIV-1 replication. Recent studies indicate that INI1 is associated with pre-integration and reverse transcription complexes that are formed upon viral entry into the target cells. INI1 also is a tumor suppressor, biallelically deleted/mutated in malignant rhabdoid tumors. We have utilized cell lines derived from the rhabdoid tumors, MON and STA-WT1, that harbor either null or truncating mutations of INI1 respectively, to assess the effect of INI1 on HIV-1 replication.

**Results:**

We found that while HIV-1 virions produced in 293T cells efficiently transduced MON and STA-WT1 cells, HIV-1 particle production was severely reduced in both of these cells. Reintroduction of INI1 into MON and STA-WT1 significantly enhanced the particle production in both cell lines. HIV-1 particles produced in MON cells were reduced for infectivity, while those produced in STA-WT1 were not. Further analysis indicated the presence of INI1 in those virions produced from STA-WT1 but not from those produced from MON cells. HIV-1 produced in MON cells were defective for synthesis of early and late reverse transcription products in the target cells. Furthermore, virions produced in MON cells were defective for exogenous reverse transcriptase activity carried out using exogenous template, primer and substrate.

**Conclusion:**

Our results suggest that INI1-deficient cells exhibit reduced particle production that can be partly enhanced by re-introduction of INI1. Infectivity of HIV-1 produced in some but not all INI1 defective cells, is affected and this defect may correlate to the lack of INI1 and/or some other proteins in these virions. The block in early events of virion produced from MON cells appears to be at the stage of reverse transcription. These studies suggest that presence of INI1 or some other host factor in virions and reverse transcription complexes may be important for early events of HIV-1 replication.

## Background

Host-virus interactions play a dynamic role during replication of all retroviruses including HIV-1 [1]. Understanding these host-virus interactions may facilitate the development of novel anti-HIV-1 strategies and efficient gene therapy vectors. One aspect of this host-virus interplay is the protein-protein interactions that exist between the viral and cellular factors [2-4]. Several viral proteins including Integrase (IN) exhibit protein-protein interactions with the host factors. IN catalyzes the insertion of viral cDNA, the product of reverse transcription, into host chromosomal DNA, by a process known as integration [5]. This process is essential for the replication of all retroviruses including HIV-1, and is a major event that leads to the development of latency [6,7]. In addition to mediating the integration reaction, IN appears to influence other processes during viral replication. IN mutations have pleiotropic effects and disrupt processes such as reverse transcription, nuclear import of pre-integration complexes, assembly and particle production [8-15]. Mechanistic basis of these pleiotropic effects is unknown, and suggests that in addition to its catalytic activity, either the intact structure of IN or its protein-protein interactions are necessary for the proper execution of various steps of HIV-1 life cycle.

Retroviral IN is expressed as a part of the Gag-Pol polyprotein, which is assembled into virions and is subsequently cleaved into individual proteins during maturation [5]. IN is carried by the virus particle into the target cells where it remains as part of the pre-integration complexes (PICs) formed subsequent to post-entry events of uncoating and reverse transcription [16]. PICs are high molecular weight nucleoprotein complexes, which in addition to retroviral cDNA contain both viral and cellular proteins [17]; [18,19].

INI1/hSNF5 and LEDGF are two host proteins that directly interact with IN [20,21]. INI1 was originally isolated as a binding partner for IN using the yeast two-hybrid system [20]. It directly interacts with IN *in vitro *and co-immunoprecipitates with Pol polyprotein *in vivo *[22]. INI1 is one of the four core components of the mammalian SWI/SNF complex that is involved in ATP-dependent chromatin remodeling [23]. The function of INI1 within this complex is not yet known, and it is thought to act as a "scaffold" bringing several components of the complex together [24]. Several recent studies indicate that INI1 and SWI/SNF complex are required for Tat-mediated transactivation of HIV-1 LTR [25-30]. INI1 is a 385 amino acid nuclear protein. It contains two highly conserved domains that are direct and imperfect repeats (Rpt) of each other and a third, fairly conserved domain termed homology region III (HRIII), at the C-terminus of the protein [31]. The Rpt domains of INI1 are involved in protein-protein interactions with both viral and cellular proteins and Rpt 1 is necessary and sufficient to bind to HIV-1 IN [31-34]. Rpt II domain of the protein contains a nuclear export signal (NES), which is masked in the full-length protein and is functional when the C-terminal domain is deleted [35].

We previously have demonstrated that an ectopically expressed dominant negative mutant of INI1, termed S6, containing the minimal IN-interaction domain potently inhibits HIV-1 assembly and particle production [22]. This inhibition was mediated by binding of S6 to IN within the context of Gag-Pol. These results suggested that the effect of dominant negative mutant of INI1 may mimic the effect of pleiotropic IN mutants and that the interaction of INI1 with Gag-Pol is required for efficient assembly and particle production. We have found that INI1 itself is specifically incorporated into HIV-1 virions and the microvescicles are devoid of this cellular protein [36]. Furthermore, incorporation of INI1 is restricted to HIV-1 and it is not found in other closely related primate lentiviruses such as HIV-2, SIV-1agm or HTLV-1 and other onco-retroviruses such as Mo-MLV [36]. This restrictive incorporation of INI1 into HIV-1 is correlated to the specific interaction of the protein with HIV-1 IN but not with other retroviral integrases. Furthermore, S6, the trasndominant mutant derived from INI1, did not affect the particle production of other related primate lentiviruses and Mo-MLV, consistent with the idea that interaction of S6 with IN is a prerequisite for its inhibitory effect. Recent immuno-precipitation studies indicated that INI1 is also a part of pre-integration and reverse transcription complexes [37]. A recent study using the siRNA mediated knock-down of INI1, specifically in the target cells (but not in the producer cells) reported that INI1 is inhibitory to early events [38]. Another study did not find inhibitory effect, but found that INI1 was not required, by using siRNA analysis [27]. Interestingly, these two reports differ in their conclusions about the role of INI1 in late events. The first study report that INI1 knock-down in the producer cells has no effect on subsequent infectivity of the virions [38]. On the other hand, the second study reported that there was inhibition of p24 production in P4 cells in which INI1 was knocked down using siRNA, indicating that INI1 may be required for late events [27]. Thus, the role of INI1 in late events in the producer cells or the role of virion-associated INI1 in early events remains unresolved.

INI1 has been demonstrated to be a tumor suppressor biallelically altered in rhabdoid tumors, an aggressive pediatric malignancy [39]. These alterations include either large changes involving the deletions spanning *INI1/hSNF5*, and/or subtle modifications involving point mutations and substitutions in this gene [40-42]. The cell lines derived from these rhabdoid tumors are INI1-defective, and hence are excellent reagents to study the effect of lack of INI1 on HIV-1 replication. Previously we have reported that HIV-1 particle production is reduced in MON cells, one of the rhabdoid cell lines carrying biallelic deletions of *INI1 *gene. Reduction in the particle production could be complemented by co-expression of INI1 in MON cells along with viral proteins [22]. However it was not clear if the inhibition of particle production is specific to MON cells or is observed in other rhabdoid cell lines. Furthermore, the stage at which the defect in infectivity of the virions produced in MON was unknown.

Since complete elimination of a host protein by RNA interference is difficult and since trace amounts of host protein may be sufficient to mediate its effect, somatic cell genetic analysis using naturally occurring cell lines harboring deletion of the gene of interest is valuable. Others and we have demonstrated that knock-out of mouse *Ini1 *by targeted disruption and homologous recombination is embryonic lethal [43-46]. In addition, deletion of *Ini1 *appears to induce apoptosis in lymphocytes in mice [47]. On the contrary, cancer cell lines with *INI1 *deletion are capable of survival presumably because of their specific cell of origin or because of the presence of additional pro-survival factors that overcome the effect of INI1 deletion. Thus, these cell lines are of interest to explore the effect of INI1 on HIV-1 replication. In this report, we have examined the effect of cellular INI1 mutations on HIV-1 replication by using two different rhabdoid cell lines, MON and STA-WT1. Our results suggest that while HIV-1 virions produced in 293T cells were able to efficiently transduce rhabdoid cells, viral replication in these cells were affected at multiple steps including particle production and subsequent reverse transcription in the next round of replication. These results suggest that INI1 may influence several steps during HIV-1 replication.

## Results

### Reduced particle production and reduced infectivity of particles produced in MON cells

We have found that INI1 is incorporated into HIV-1 virions and HIV-1 based vectors [22]. To examine the influence of either cellular INI1 present in the target cells or the virally incorporated INI1 during HIV-1 infection, we tested the efficacy of infecting INI1-defective rhabdoid cells, MON and STA-WT1, by the VSV_G _pseudotyped virions produced in 293T cells, using GFP as the marker. We found that HIV-1 based vectors transduced MON cells lines as efficiently as that of the 293T control cells (data not shown).

**Figure 1:**
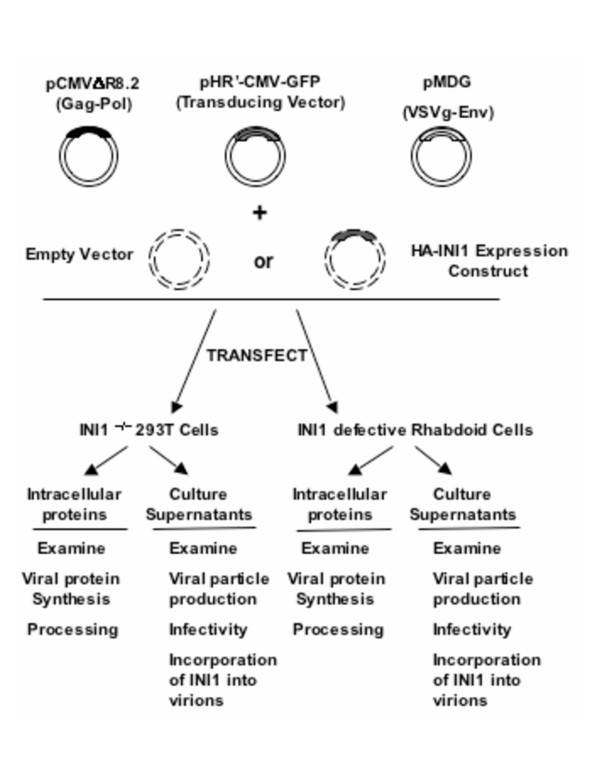
**Experimental Strategy used to analyze steps of HIV-1 replication in INI-deficient cells**. Viral vectors pCMVΔR8.2, pMDG and pHR'-CMV-GFP were co-transfected with or without plasmid expressing HA-INI1 into 293T cells and INI-defective cells. Culture supernatants and cell lysates were examined for levels of the virus particle production and infectivity of the produced virions, incorporation of viral proteins and INI1, viral protein synthesis and efficiency of processing.

We next used the strategy described in Figure 1 to determine replication of HIV-1 in rhabdoid cells containing INI1 mutations. We first used MON cells that carry biallelic deletions of *INI1 *gene along with 293T cells. Both cell lines were transiently transfected with three-plasmid-based vectors containing CMV-GFP as a marker and pseudotyped with VSV_G _envelope. Viral protein synthesis and particle production in the cells were monitored by p24 ELISA. The HIV-1 based vectors produced in MON and 293T cells were then analyzed for their ability to infect both cell types by utilizing GFP as reporter.

**Figure 2:**
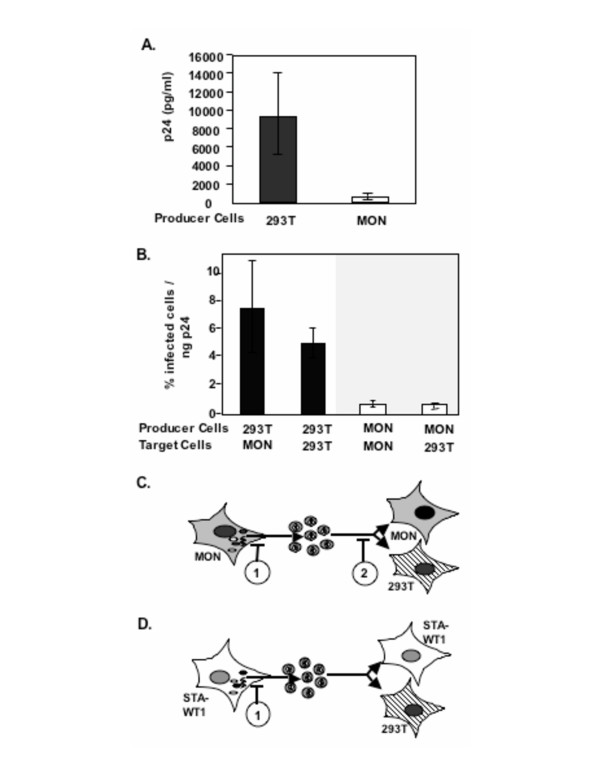
**Analysis of particle production and infectivity in MON cells: A**. Graphic representation of p24 antigen release (pg/ml) in the culture supernatants of 293T (■) and MON (□) cells transfected with three plasmid based HIV-1 vectors (average of 3 independent experiments). **B**. Graphic representation of infectivity of virions, normalized for p24, produced in either 293T (■), or MON (□). Both 293T and MON cells were infected with these two sets of virions (average of 3 independent experiments). **C**. Schematic representation of 2 blocks in HIV-1 replication in MON cells. 1^st ^block exists at the level of virus particle production and the 2^nd ^block exists at the level of infectivity. **D**. Schematic representation of a block in HIV-1 replication in STA-WT1 cells at the level of virus particle production.

The above analysis confirmed our previous report of greater than 10-fold reduction in the amount of particle production in MON cells as compared to that of 293T cells (Figure 2A). Culture supernatants from 293T and MON cells, normalized for p24, were used to infect both cell types, and the percentage of infection was determined as before (Figure 2B). Results of these experiments indicated that while virions produced in the 293T cells were able to infect both 293T and MON cells, virions produced in MON cells exhibited a drastic reduction in infectivity on both 293T and MON cells (Figure 2B). The above results suggested that there are two blocks to HIV-1 replication in MON cells (Figure 2C). The first block results in 10–20 fold reduction in the virus particle production in MON, and the second block leads to reduced infectivity of virions by about 10 to 15 fold as compared to that produced in 293T cells.

### INI1/hSNF5 complements the defect in particle production but not the defect in infectivity

**Figure 3:**
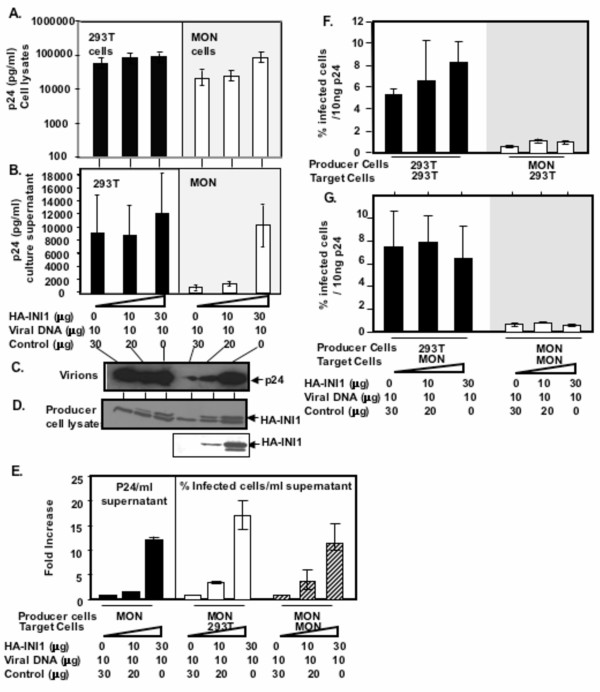
**INI1 complements defect in the virus particle production in the MON cells in a dose-dependent manner. A**. Graphic representation of intracellular p24 (pg/ml) in the 293T (■), and MON (□) cells in the presence of increasing concentrations of HA-INI1 (average of 3 independent experiments). **B**. Graphic representation of particle associated p24 (pg/ml) in the 293T (■), and MON (□) cells in the presence of increasing concentrations of HA-INI1 (average of 3 independent experiments). **C**. Immunoblot analysis of concentrated virions produced in 293T and MON cells in the presence of increasing concentrations of INI1 using α-p24 antibody. **D**. Immunoblot analysis of relative amounts of transfected HA-INI1 in the producer cells. The upper panel was probed with α-HA and the lower panel was probed with affinity purified α-INI1 antibodies. **E**. Graphic representation of fold increase in the virus particle production (■) and infectivity of virions produced in MON cells over 293T (□) and MON(....) cells. (**F and G**). Graphic representation of % infectivity of normalized virus particles over 293T cells (F) and MON cells (G). Virions were produced in 293T (■) or MON (□) cells in the presence of increasing concentrations of INI1 (average of 3 independent experiments).

To determine if the efficiency of particle production or the efficiency of transduction/infectivity can be complemented by re-expression of INI1, we co-transfected increasing concentrations of a plasmid expressing Heamagglutinin (HA) tagged INI1/hSNF5, along with constant amounts of three plasmid-based-vectors into both MON and 293T cells. Viral protein synthesis and particle production, were monitored by P24 ELISA. Results of these analyses indicated that there was an increase (about 3 fold, ** p = 0.05 for 293T and *p *= 0.024 for MON) in the intracellular levels of p24 in both MON and 293T cells in the presence of INI1 (Figure 3A). However, the presence of INI1 in MON cells resulted in further increase **(**about 10 fold *p = 0.03) in particle production as compared to that in 293T cells (*p *= 0.07, not significant) (Figure 3B,C). Immunoblot analysis of the producer cell lysates indicated that comparable levels of HA-INI1 were expressed in both cell types (Figure 3D). These results indicate that INI1/hSNF5 is able to increase the efficiency of particle production in MON cells, in part by increasing the intracellular viral proteins and in part by increasing viral release (Figure 3E). These results are consistent with our previous report, where we indicated that with re-introduction of INI1, there was increase in viral particle production as measured by p24 and an increase in number of infectious units [22].

Previously we measured infectivity per ml of the supernatant regardless of the amount of p24 present in the supernatants in the presence or absence of INI1[22]. Here we examined the infectivity of virions produced in the presence or absence of INI1 after normalizing the culture supernatants for the levels of p24. The results indicated that irrespective of the presence of INI1/hSNF5, the infectivity per unit amount of p24 of virions produced in MON cells remained low (about 10 fold low) as compared to that of virions produced in 293T cells (Figure 3F and 3G). The results were similar whether the target cells used for transduction were 293T (Figure 3F) or MON (Figure 3G) cells. These results suggested that re-expression of INI1 in MON cells increased the viral particle production, as measured by p24, and number of infectious particles, as measured by infectivity per volume. However, the infectious units produced in MON per given amount of p24 remained low. These results are consistent with the hypothesis that there are two blocks for HIV-1 replication in MON cells as illustrated in the Figure 2C.

### IN and INI1 but not other components of SWI/SNF complex are required for efficient particle production

**Figure 4:**
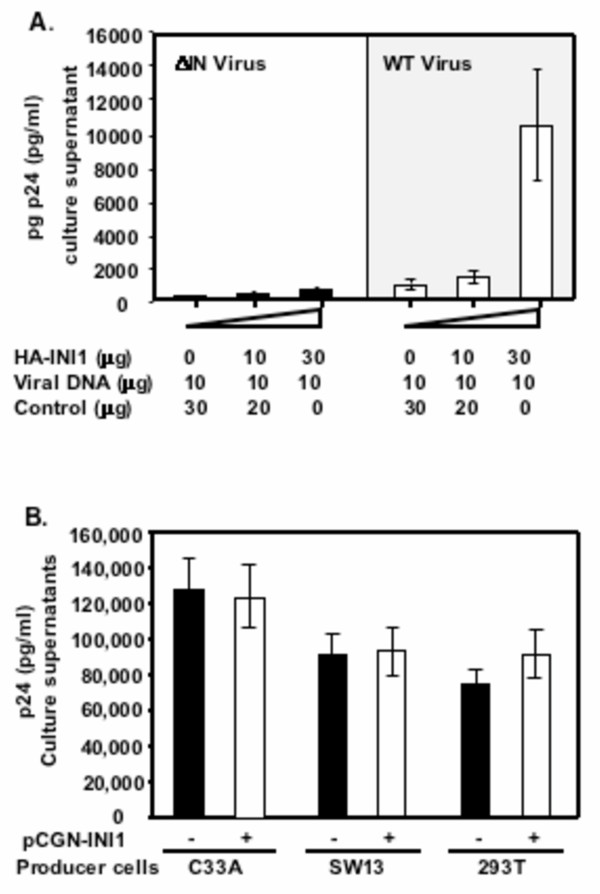
**IN and INI1 but not the components of the SWI/SNF complex are required for efficient HIV-1 particle production. A**. IN is required for the complementation of particle production by INI1. Graphic representation of particle-associated p24 antigen (pg/ml) in the MON culture supernatants transfected with either ΔIN Gag-Pol **(■) **or wild type Gag-Pol (□) along with increasing concentrations of INI1 (average of 2 independent experiments). **B**. The ATPase components of SWI/SNF complex are not required for HIV-1 particle production. Graphic representation of particle associated p24 released from SW13 and C33A, the two BRG1 and hBRM-dificient cells and 293T cells in the absence (■) or presence (□) of transfected HA-INI1 (average of 3 independent experiments).

The above results suggest that INI1 has a role in late events of HIV-1 replication. To determine if IN-INI1 interaction is required for the increase in the viral particle production, we tested the effect of INI1 on particle production when IN was deleted from Gag-Pol. We surmised that if INI1 effect is indirect, then presence or absence of IN should yield the same result of increase in particle production. We inserted a stop codon at the beginning of IN in pCMV-ΔR8.2 plasmid to generate pCMV-ΔR8.2ΔIN, and co-transfected this construct with plasmids encoding envelope and transducing vector along with increasing concentrations of INI1/hSNF5 expression plasmid into MON cells. Particle production was monitored as before by p24 ELISA (Figure 4A). Results of these experiments indicated that while there was a 10–15 fold increase in particle production in the case of wild type virus, in the absence of IN there was no significant increase in the p24 release with ΔIN virus, suggesting that IN-INI1 interaction is necessary for this effect.

INI1/hSNF5 is a component of the mammalian SWI/SNF complex. To determine if other components of this complex are necessary for HIV-1 particle production, we transfected HIV-1 DNA into C33A and SW13 cells that are defective for BRG1 and BRM, the ATPase components of the SWI/SNF complex. We found that there was no defect in p24 production in these cells as compared to that of 293T cells (Figure 4B). Thus it appears that the ATPase components of the SWI/SNF complex, BRG1 or hBRM, and perhaps the intact SWI/SNF complex are not required for the efficient HIV-1 particle production. This presents an additional evidence to suggest that INI1-induced increase in particle production in MON cells is not due to the increase in viral transcription, since the activation of transcription by INI1 is dependent on the ATPase components of the SWI/SNF complex ([33] and unpublished data).

### Characterization of HIV-1 particle production in STA-WT1 rhabdoid cell line

To determine if the reduction in particle production we observe in MON cells is seen in other INI1-defective cell lines, we tested the replication of HIV-1 in another rhabdoid cell line, STA-WT1, where one of the alleles of *INI1 *gene is deleted and the other allele harbors a single nucleotide deletion (delG950), leading to the expression of a truncated protein (ΔINI1, aa 1–319). This truncated INI1 protein retains two highly conserved Rpt domains that include the IN-binding region, and is mis-localized in the cytoplasm [35].

**Figure 5:**
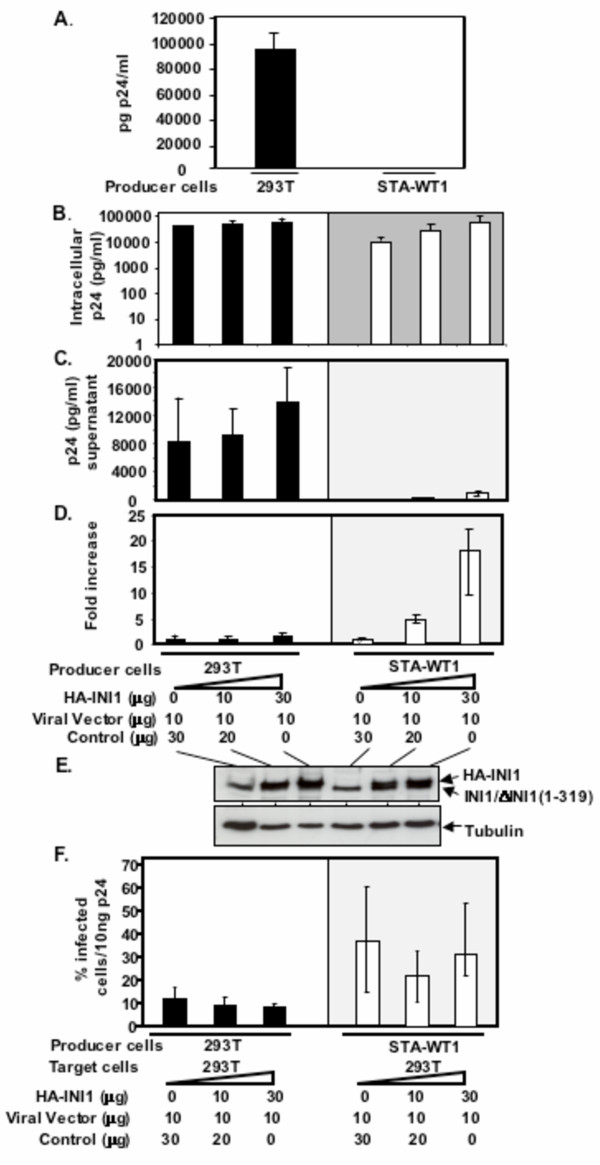
**Analysis of HIV-1 particle production and infectivity of the virions from STA-WT1 cells. A**. Graphic representation of virion-associated p24 antigen (pg/ml) in the culture supernatants of 293T (■) and STA-WT1 (□) cells (average of 3 independent experiments). **B**. Graphic representation of intracellular p24 (pg/ml) in the 293T (■), and STA-WT1 (□) cells in the presence of increasing concentrations of HA-INI1 (average of 3 independent experiments). **C**. Graphic representation of virion-associated p24 antigen (pg/ml) in the culture supernatants of 293T (■) and STA-WT1 (□) cells, co-transfected with the viral vectors and increasing amounts of INI1 (average of 3 independent experiments). **D**. The above results (B) represented as relative fold increase in particle production. **E**. Immunoblot analysis of relative amounts of transfected INI1 in 293T and STA-WT1 producer cells (top panel), using α-INI1 antibody. The same blot was reprobed with α-tubulin antibody as a loading control (bottom panel). **F**. Graphic representation of the % infectivity of particles normalized for p24, produced in 293T (■) or STA-WT1 (□) cells.

As before, we used single cycle infection assays to determine the ability of STA-WT1 cells to support VSV_G _pseudotyped HIV-1 particle production. We found that particle production was dramatically decreased in these cells (about 100 fold) as compared to that of 293T cells (Figure 5A). This defect in virus particle production is similar to that observed in MON cells and hence we next examined if full length INI1 could complement this defect. Similar to that of MON cells, there was an increase (about 3–5 fold) in intra-cellular levels of p24 in the presence of INI1 (Figure 5B). Consistent with the finding in MON cells, co-transfection of INI1 resulted in a dose-dependent increase in particle production. We found that the highest concentration of INI1 tested resulted in about 20-fold increase in particle production, thus leading to a significant but partial complementation (Figure 5C, D). Western analysis using α-INI1 antibodies indicated the presence of both endogenous and transfected INI1 (Figrue 5E). Thus these results corroborate our previous finding that presence of full length INI1 serves to increase HIV-1 particle production in two different cell lines.

To determine the infectivity of particles produced from STA-WT1 cells we infected 293T cells with HIV-1 vector-containing culture supernatants normalized for p24 levels. We found that, even though the amount of particles released in STA-WT1 in the absence of transfected full length INI1 was lower as compared to that released from the 293T cells, their infectivity per unit amount of p24 was similar to or even higher than those produced in 293T cells (Figure 5F). Thus, it appears that although there was a definitive block in particle release, there was no block in the infectivity of the particles produced from STA-WT1 cells (Figure 2D). These results indicated that there is a qualitative difference in the infectivity of particles produced from two rhabdoid cells, MON and STA-WT1.

### Absence of INI1/hSNF5 incorporation into the virions produced from MON

The above set of results presented us with an interesting paradox. One common feature, that we observed in both MON and STA-WT1 cells, was that particle production was low in these cells and re-introduction of INI1 increased the particle production significantly up to 20 fold. However, while virions produced in MON cells were defective for infection, those produced in STA-WT1 cells were infectious. These results indicate either one of the following possibilities. (i) INI1 in the virions is not necessary for infectivity; (ii) some cellular factor, which is present in STA-WT1 but not in MON, is necessary for infectivity; (iii) some cellular inhibitory factor, present in MON but absent in STA-WT1 cells, reduce the infectivity of particles produced from MON. The main difference between these two cell lines is the complete lack of INI1 in MON, and the presence of a truncated, cytoplasmic form of INI1 (ΔINI1, aa 1–319) in STA-WT1 cells. This cytoplasmic fragment of INI1 retains the two Rpt domains and is potentially available for incorporation into the virions [35]. Therefore, it is possible that the cytoplasmically localized INI1 in STA-WT1 cells is nevertheless packaged into the HIV-1 particles and is sufficient to mediate early events of HIV-1 replication.

**Figure 6:**
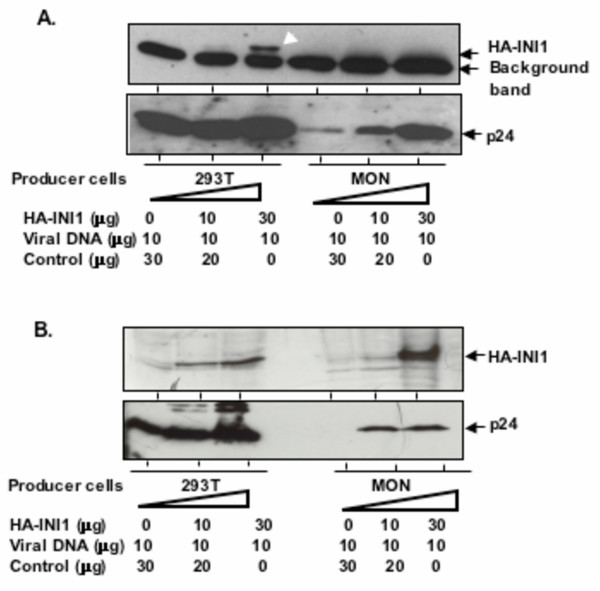
**Analysis of INI1 incorporation into virus particles. A**. Virions produced in MON cells fail to efficiently incorporate INI1. Immunoblot analysis of highly concentrated and subtilisin treated virions produced from 293T and MON cells in the presence of increasing concentrations of INI1, using α-HA antibody (top panel) followed by α-p24 antibody (bottom panels). Bottom band in all the lanes of the top panel is due to nonspecific binding of the antibody. **B**. Virions produced in STA-WT1 cells efficiently incorporate INI1. Immunoblot analysis of highly concentrated and subtilisin treated virions produced from 293T and STA-WT1 cells in the presence of increasing concentrations of INI1, using α-INI1 antibody (top panel) followed by α-p24 antibody (bottom panels).

To test the above hypothesis, we prepared purified and highly concentrated virions produced from 293T, MON and STA-WT1 in the presence or absence of transfected HA-INI1, treated them with subtilisin, and subjected them to immunoblot analysis. As expected, we noticed a band of correct molecular weight corresponding to HA-INI1 in virions produced from 293T cells (Figure 6A). However, the virions produced from MON cells, even in the presence of high level of INI1, exhibited very little to no HA-INI1 incorporation (Figure 6A, compare lane 3 to lane 6). This lack of incorporation of INI1/hSNF5 was not due to the lack of expression of INI1 in these cells (Figure 3D). These results indicated that virions produced in MON cells show reduced or lack of INI1/hSNF5 incorporation into the virus. At this point we do not know the reason for this lack of INI1 incorporation. Unfortunately, we were unable to express Vpr-fusion of INI1 in MON cells as Vpr expression itself was toxic to these cells (data not shown).

We next examined the incorporation of INI1 into virions in the STA-WT1 cells. Production of low amounts of virions from STA-WT1 cells in the absence of INI1 made it difficult to detect endogenous truncated INI1 in the virions by immunoblot analysis. This is because INI1 is incorporated in a stoichiometric ratio of 2:1 IN:INI1 [36] and there are about 50 molecules of INI1 per virions. This low amount per virion, combined with the low titer of INI1 antibody and very low amounts of particle production, makes it prohibitive to detect endogenous INI1 in STA-WT1 produced virions. However, sufficient quantities of virions were produced in the presence of HA-INI1 and hence we concentrated these virions and subjected them for immunoblot analysis. These results indicated that virions produced in STA-WT1 cells efficiently incorporated HA-INI1 (Figure 6B). Lack of INI1 in virions produced from MON and presence of INI1 in virions produced from STA-WT1 cells, and their relative infectivity, is in support of the hypothesis that INI1 itself or INI1 associated proteins present in the virions may influence the efficiency of infectivity of these particles.

### Virions produced in MON cells exhibit defect in reverse transcriptase activity

**Figure 7:**
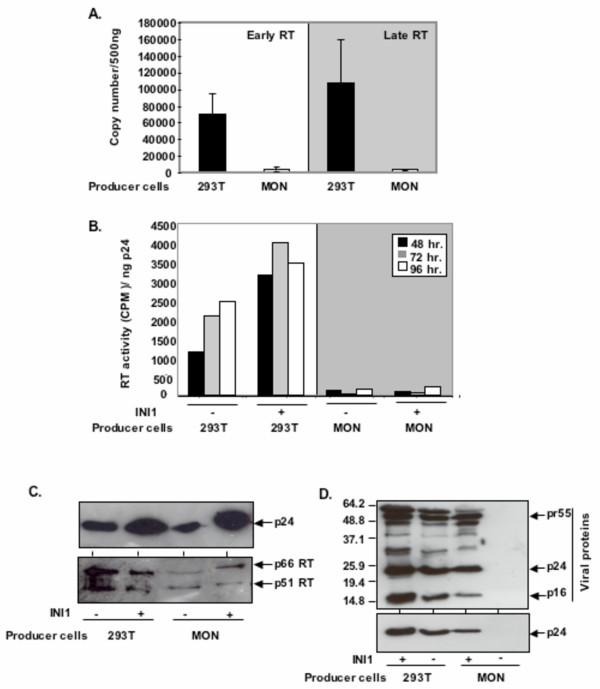
**Virions produced in MON cells are defective for early reverse transcription. A**. Graphic representation of copy number of early and late reverse transcription products formed upon infection of 293T cells. Equal quantities of virions produced in 293T (■) and MON (□) cells were used for infection and the copy numbers of RT products were determined by quantitative real time PCR. **B**. Reverse transcriptase activity of virions produced in 293T and MON cells. Virions collected at various times after transfection were concentrated and quantitated by p24 ELISA. Normalized amounts of virins were subjected to reverse transcriptase analysis. The graph represents CPM incorporated per ng of p24. **C**. and **D**. Analysis of concentrated virion proteins produced in MON cells in the presence of INI1. Immunoblot analysis of the virions produced in 293T and MON cells in presence or absence of INI1, by using α-p24 (C, top panels and D, bottom panel), α-RT (C, bottom panel), and α-hIgG from a patient serum (D, top panel). ...

We next characterized the exact stage of defect in infectivity of virions produced from the MON cells. It is unlikely that the defect in early events was at the entry step as the particles are pseudotyped with VSV_G_. Since INI1 has been demonstrated to be present in both cytoplasmic and nuclear reverse transcription complexes, the defect could be at any step after the entry, including uncoating, reverse transcription, formation of PICs, nuclear localization and/or integration. To determine the step at which the replication of virions produced in the MON virus is blocked in vivo, we carried out quantitative real time PCR to determine the amount of newly synthesized viral DNA in infected cells. Our results indicated that while virions produced in 293T cells exhibited the robust amount of viral DNA synthesized due to reverse transcription, the virions produced in MON exhibited a drastic reduction in both early (minus strand strong stop DNA) and late (Gag) viral DNA synthesis in the infected cells (Figure 7A).

We also tested the ability of the particles produced in MON cells to mediate reverse transcriptase activity in vitro using poly rA-oligo dT template-primer (Figure 7B). The RT activity was normalized as CPM incorporated per ng of virus associated p24. The results of these experiments indicated that while the virions produced in 293T cells demonstrated slightly increased RT activity in the presence of INI1, virions produced in MON were defective for reverse transcriptase activity (Figure 7B). These results suggest that virions produced in MON cells are blocked at reverse transcription step in the target cells.

One possibility we considered is that the virions produced in MON may be specifically defective for incorporation of Gag-Pol but not Gag molecules, as INI1 binds to IN, and that the lack of Gag-Pol incorporation could explain the lack of reverse transcriptase activity in these virions. To test this possibility, we subjected purified and concentrated virions, produced in MON cells in the presence and absence of INI1, to immunoblot analyses with antibodies against RT, p24 and anti-HIV-1 antibodies derived from patient serum. As expected, there was a significant increase in the levels of p24 in the culture supernatants of MON cells when INI1 was expressed in the producer cells. Furthermore, the purified virions produced in these cells demonstrated the presence of all the viral proteins, although there were some minor differences in the partially processed viral proteins (Figure 7C, D). These results suggested that perhaps INI1 and/or other host proteins are necessary for a step before or during the early events of reverse transcription.

...

## Discussion

In this report, we demonstrated that replication of HIV-1 is affected at multiple steps in cell lines defective for the integrase binding host factor INI1/hSNF5. We used two rhabdoid cell lines, MON and STA-WT1 defective for *INI1/hSNF5 *gene in our studies. While MON cells harbor complete deletion of *INI1 *gene, STA-WT1 cells harbor a single point mutation in the gene resulting in the expression of a cytoplasmically localized truncated protein [35]. Our results demonstrate that rhabdoid cells are efficiently infected by HIV-1 indicating that expression of a full length INI1 in the target cells is not required. These results are not surprising, as we have previously demonstrated that INI1 is specifically incorporated into HIV-1 virions with an IN:INI1 ratio of approximately 2:1 [36]. Therefore, it is possible that INI1 brought into the target cells by the virions may be sufficient to support early events of HIV-1 replication. Furthermore, recent reports indicated that siRNA (small interfering RNA) mediated knock-down of INI1 in the target cells did not inhibit HIV-1 infection, or enhanced the infectivity to some degree [27,38]. These results are consistent with our results that MON and STA-WT1 cells are readily transduced by the HIV-1 based vectors. Maroun et al also reported that knock-down of INI1 in the producer cells did not affect the infectivity. However, these later experiments need to be further substantiated, as no data was provided in this report that demonstrated the lack of INI1 in the virion particles produced from the INI1 knock-down cells.

Our analysis also indicated that both MON and STA-WT1 cells exhibit reduced particle production, which is enhanced/complemented by reintroduction of full length INI1 into these cells. Interestingly this enhancement was not efficient for virions deleted of IN indicating that perhaps, binding of IN to INI1 is required for efficient particle production. Furthermore, the cell lines defective for ATPase component of the SWI/SNF complex such as BRG1 and BRM are not defective for particle production. These results suggest that INI1 alone, but not the SWI/SNF complex is required for HIV-1 particle production. It is not clear how INI1 facilitates late events at this point. But our results suggest that INI1 affects late events at two stages. On the one hand it enhances the levels of viral proteins within the rhabdoid cells and on the other hand it also increases virus particle production. INI1 could stabilize and direct the viral proteins into specific compartments or it may be required to overcome some host inhibitory factors that could block these events in these cells.

It is intriguing that particles produced in MON cells exhibited reduction in infectivity as compared to those produced in either 293T cells or STA-WT1 cells, when normalized for p24. Both MON and STA-WT1 cells are rhabdoid cells and while MON cells completely lack INI1, STA-WT1 cells possesses a truncated version of INI1. Examination of the particles produced in these two cell lines indicate that while INI1 was efficiently incorporated into the particles produced in STA-WT1, it was not incorporated into the particles produced in MON. Thus, infectivity of HIV-1 particles correlated with the ability to incorporate INI1. These results suggested that presence of INI1 in the producer cells and its incorporation into the virions may enhance the particle production and infectivity of HIV-1. However, at this point we cannot rule out the possibility that the reduced infectivity of the virions could either be due to the lack of other INI1-associated proteins or another host protein unrelated to INI1 in MON cells. It remains to be determined as to what causes the block in incorporation of INI1 into HIV-1 particles in MON cells.

It is interesting to note that the amount of particle production is much less in STA-WT1 as compared to that of 293T or MON cells even in the presence of high amount of INI1. Furthermore, particles produced in STA-WT1 cells are more infectious than those produced in 293T cells. One possible explanation for the above observation is the presence of truncated INI1 (ΔINI1, aa 1–319) in STA-WT1 cells. This mutant retains integrase binding Rpt1 domain. We have previously demonstrated that this truncated mutant is localized in the cytoplasm in STA-WT1 cell lines [35]. We believe that presence of this mutant makes it harder for the particle release, as it acts as a dominant negative mutant, and that the wild type INI1 is able only to partially complement this defect.

The defect in infectivity of virions produced in the MON cells appears to be due to a block in reverse transcription in the target cells. This block in reverse transcriptase activity was not complemented by INI1, so is infectivity and is consistent with the lack of INI1 in the particles. These results although unexpected, are not unprecedented. Mutants of IN have been shown to affect reverse transcription and this function has been ascribed to the direct binding of IN to RT [9-11]. At this point, mutants of RT that are solely defective for IN binding and show reduced activity have not been isolated, to unequivocally demonstrate that direct binding of these two proteins alone is sufficient to modulate RT activity. Our results that lack of INI1 in the virions make the particles defective for reverse transcriptase activity, raises the possibility that INI1 or another component that is missing in MON may be required for efficient reverse transcriptase activity. The fact that INI1 indeed is a part of the reverse transcription complexes in the target cells, further lends support to the notion that INI1 may somehow be involved in early reverse transcription [37]. Further experiments are needed to elucidate the mechanism by which particles produced in MON are defective for reverse transcriptase activity.

## Conclusion

Our results indicate that there is a decreased HIV-1 particle production in cell lines defective for INI1. Reintroduction of INI1 results in increase in particle production in these cells, suggesting that INI1 in the producer cells is necessary for efficient particle production. Furthermore, while HIV-1 virions produced in MON cells are less infectious, those produced from STA-WT1 were more infectious. Analyses of the virions produced in these cells indicate that while particles produced from MON cells failed to incorporate INI1, those produced from 293T efficiently incorporated INI1. Particles produced from MON cells lacking INI1 were defective for carrying out early and late reverse transcription in target cells. Furthermore, reverse transcriptase activity of the virions produced in MON was defective. These results suggest that defect in INI1 or some other host function of rhabdoid cells modulate the efficiency of HIV-1 replication.

## Methods

### Virus particle production

293T, MON and STA-WT1 cells were co-transfected with a total of either 10 or 20 μg of DNA containing pHR'-CMV-GFP, pCMV-ΔR8.2 and pMDG (expressing VSV_G _envelope) in a 2:1:1 ratio respectively, using the calcium phosphate transfection kit (Specialty Media). For INI1 complementation studies, cells were co-transfected with 10 μg of the above plasmids along with increasing amounts of pCGN-INI1 (expressing HA-INI1), keeping the total amount of transfected DNA constant at 40 μg by using pCDNA. Culture supernatants were collected from transfected cells 48 hrs post-media change, treated with 20 U/ml of DNAseA [36], and subjected to p24 ELISA (DuPont).

### Analyzing the infectivity of virions

About 10^5 ^cells seeded in six well plates were infected by the normalized amounts of virions, and percentage of GFP-positive cells was determined by FACS (Fluorescent activated cell sorter), five days post-infection. Quantitation of viral proteins was determined by using p24 ELISA using standard methods.

### Subtilisin treatment of purified HIV-1 virions

Virions purified and concentrated by sucrose density gradient centrifugation were treated with subtilisin as described [36]. The treated virion pellets were resuspended in the lysis buffer (25 mM Tris-Cl, pH 7.5, 50 mM KCl, 0.025% Triton X-100, 50% glycerol) and mixed with 2x loading buffer (2 × = 0.25 M Tris-Cl pH 6.8, 8% SDS, 40% glycerol, 3.6% β-mercaptoethanol, 0.005% bromophenol blue).

### Antibodies

Immunoblot analyses were carried out by chemiluminescence methods by using the antibodies: (i) monoclonal α-IN antibodies (hybridoma clone #8E5-1F9); (ii) affinity purified polyclonal α-INI1 antibody, INI1-PB3 [22]; (iii) goat polyclonal α-p24 antibody (Goat#81, Bleed # 001090); (iv) monoclonal α-Tubulin antibody (Sigma, Catalogue # T5168); (v) polyclonal α-HA antibody (Santa Cruz, catalogue# SC-805); (vi) patient serum containing α- HIV-1 antibody (AIDS Reagent Program, catalogue # 3957); and (vii)monoclonal α-RT antibody (clone 8C4D7).

### Quantitative Real-Time PCR

Normalized, DNase-1 treated virions were used to infect 1.0 × 10^5 ^293T cells in 6-well plates for 2 hours, then washed with PBS, and placed into fresh DMEM for additional 4 hours. Genomic DNA was isolated at 6 hr. post-infection using DNeasy kit (Qiagen). Early HIV-1 reverse transcripts were quantitated using primers ert2f, ert2r and the ERT2 probe [48]. Late HIV-1 reverse transcripts were quantitated with primers MH531, MH532, and the probe LRT-P [49]. Each 20 μl reaction contained 1x Taqman universal master mix (Applied Biosystems), 300 nM primers, 100 nM Probe, and 500 ng of genomic DNA. PCR conditions were as described before [49]. Reactions were analyzed in triplicates using the ABI Prism 7700 sequence detection system (Applied Biosystems).

### RT assay

The virions were precipitated by adding PEG and were subjected to RT assay as previously described using poly rA-oligo dT template-primers and α-P^32 ^dTTP [36].

## Competing interests

The authors declare that there are no competing interests.

## Authors' contributions

MS: Designed and optimized methods and performed majority of the experiments and contributed to the writing of the manuscript.

EY: Performed part of the experiments.

XW: Performed part of the experiments and assisted MS in rest of the experiments.

GK: Designed, supervised, interpreted and oversaw all the experiments, wrote the manuscript with the assistance of MS.
